# Automated Analysis of the Foveal Avascular Zone in Optical Coherence Tomography Angiography Before and After Phacoemulsification

**DOI:** 10.3390/jcm14217674

**Published:** 2025-10-29

**Authors:** María S. Pighin, Evangelos Tsiroukis, Agniezska Dyrda, Ignasi Jürgens

**Affiliations:** Institut Català de Retina, 08022 Barcelona, Spain; evangelostsiroukis@gmail.com (E.T.); agnieszkaannadyrda@wp.pl (A.D.); ignasi.jurgens@icrcat.com (I.J.)

**Keywords:** retinal imaging, foveal avascular zone, machine learning, optical coherence tomography angiography, quantitative analysis, Waikato environment for knowledge analysis (WEKA)

## Abstract

**Objective:** This study aimed to compare two methods for measuring the foveal avascular zone (FAZ) before and after phacoemulsification: a script-based semiautomated algorithm and a machine learning (ML)-based semiautomated algorithm. **Methods:** Optical coherence tomography angiography (OCTA) images were obtained with a Spectralis OCTA system (Heidelberg Engineering, Germany) preoperatively and in three postoperative visits. The FAZ was measured using both methods. **Results:** The study analyzed 708 OCTA scans from 59 eyes. Correlation analyses showed strong agreement between the semiautomated script-based and ML-based methods in the three plexuses, with Pearson correlation coefficients of *r* = 0.836 (95% CI: 0.74–0.89), *r* = 0.646 (95% CI: 0.45–0.78), and *r* = 0.861 (95% CI: 0.78–0.92), respectively (*p* < 0.0001 for all). In longitudinal analysis, the FAZ in the SVP decreased significantly after phacoemulsification at 1 and 2 months postoperatively with both the script-based method (*p* = 0.017 and *p* = 0.039) and the ML-based method (*p* < 0.0001 and *p* = 0.004). **Conclusions:** Our findings suggest that the ML-based approach is a reliable method for measuring the FAZ on OCTA, comparable to the semiautomated script-based algorithm, and may serve as a practical alternative. Moreover, a significant reduction in FAZ within the SVP was observed two months after phacoemulsification.

## 1. Introduction

Optical coherence tomography (OCT) has become a standard diagnostic tool for monitoring therapeutic efficacy in patients with macular pathology. More recently, optical coherence tomography angiography (OCTA) has gained increasing use in clinical practice, providing a noninvasive evaluation of retinal and choroidal microvasculature. In contrast to fluorescein angiography, OCTA is dye-free and enables depth-resolved visualization of vascular plexuses [1]. Fluorescein angiography, by comparison, provides only two-dimensional images with overlapping plexuses and requires intravenous dye administration, which may be associated with adverse events [2].

Although quantitative analysis of OCTA images is limited to specific commercial devices [3], open-source software such as Fiji (ImageJ2, version 2.16.0, Madison, WI, USA) can reproduce these analyses in a reproducible and comparable manner. This platform is an image-processing package specifically designed for scientific applications and is widely adopted in biomedical research [4]. Although foveal avascular zone (FAZ) characterization using OCTA has been extensively studied, no standardized approach has yet been established [5]. OCTA devices lacking built-in quantification tools may benefit from third-party software solutions, including artificial intelligence, to achieve accurate FAZ measurements. Such strategies could contribute to the standardization of OCTA parameters. In this study, we trained the Trainable Weka Segmentation plugin, a Fiji-based machine learning (ML) tool [6], and compared its performance with a script specifically designed for FAZ analysis in Spectralis OCTA images.

Moreover, evaluating the foveal avascular zone (FAZ) before and after cataract surgery enables us to investigate the physiological impact of phacoemulsification on macular microvasculature. This comparison also serves as a test case for assessing whether semiautomated image analysis tools can reliably capture longitudinal vascular changes in a real-world clinical setting.

## 2. Materials and Methods

This study included human participants and was approved by the local Ethics Committee (Idcsalud Catalunya; ID 2017/62-OFT-ICR) and adhered to the tenets of the Declaration of Helsinki. Written informed consent was obtained from all participants. Patients undergoing phacoemulsification between January 2018 and January 2020 were enrolled. Patients were excluded if they had any retinal vascular disease, diabetic retinopathy, glaucoma, or axial length ≥ 26 mm. To minimize the impact of media opacity, only patients with nuclear opalescence grade ≤ 3 according to the LOCS III classification were included.

OCTA images were acquired with the Spectralis OCTA system (Heidelberg Engineering, Heidelberg, Germany) at baseline and at 1, 4, and 8 weeks postoperatively. The acquisition protocol consisted of 512 high-resolution B-scans covering a 3 × 3 mm area centered on the fovea. The Spectralis default segmentation software automatically identified three vascular plexuses: the superficial vascular plexus (SVP), extending from the nerve fiber layer to the inner plexiform layer; the intermediate vascular plexus (IVP), from the inner plexiform layer to the inner nuclear layer; and the deep vascular plexus (DVP), from the inner nuclear layer to the outer plexiform layer [7]. All scans were obtained under standardized pharmacologic mydriasis (1% tropicamide and 2.5% phenylephrine).

OCT images were classified according to Huang [8], and poor-quality scans (quality index ≤15) were excluded. Images with motion artifacts, such as horizontal lines across the central field, were also excluded. Although the algorithms used (script-based and Weka-based) do not include intrinsic compensation for media opacity, all analyses were conducted on images that passed this quality threshold explained above, thereby minimizing the impact of cataract-related image degradation.

Data were adjusted according to the methods of Sampson and Linderman [9,10]. To account for ocular magnification due to interindividual variation in axial length (AL), we applied image scaling correction to all FAZ area measurements using the modified Bennett formula. The corrected image scale was calculated by multiplying the nominal scale (based on a 3 × 3 mm field and 510 pixels) by the ratio of each subject’s measured AL to the reference AL assumed by the OCTA system (23.95 mm). The resulting image scale was then squared and used to convert pixel-based FAZ areas to real-world retinal dimensions in mm^2^. In total, 708 OCTA images were obtained and converted into Tag Image File (.tif) format for analysis in Fiji software.

This was a non-blinded study. Due to the nature of the intervention (phacoemulsification) and the clinical workflow, neither the patients nor the examiners were blinded to the pre- or postoperative status. OCTA image acquisition and analysis were conducted without masking, reflecting routine clinical conditions.

The sample size was determined based on Brizzi [7], a previously published study that established the number of patients required to detect intraretinal cysts through interferometric methods. Given our working hypothesis—namely, that OCTA provides comparable structural information to fluorescein angiography but in a non-invasive manner—this estimate was used as the reference for recruitment. Fluorescein angiography was not performed due to its invasive nature and was outside the scope of this non-interventional study.

**Figure 1:**
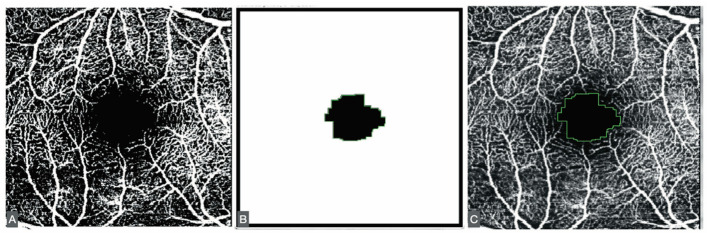
(**A**) Otsu binarization of the SVP. (**B**) The results of the ImageJ script. (**C**) Fit of the result of the script in the 8-bit former image.

The FAZ was measured using a script adapted from Ishii [11]. Minor modifications were introduced, including adjustment of image size and the application of Otsu binarization [12], a local/global thresholding method that reduces artifacts compared with other binarization algorithms (Figure 1).

The script was set as follows:

run(“8-bit”);

makeRectangle (127, 127, 512, 512)

run(“Crop”);

run(“Size...”, “width = 270 height = 270 constrain average interpolation = Bilinear”);

run(“Auto Threshold”, “method = Otsu white”);

setOption(“BlackBackground”, true); run(“Skeletonize”);

run(“Dilate”);

run(“Dilate”);

run(“Dilate”);

run(“Dilate”);

run(“Dilate”);

run(“Dilate”);

run(“Dilate”);

run(“Dilate”);

run(“Erode”);

run(“Erode”);

run(“Erode”);

run(“Erode”);

run(“Size...”, “width = 512 height = 512 constrain average interpolation = Bilinear”);

doWand(255, 255);

roiManager(“Add”);

In addition, FAZ measurements were obtained using the Trainable Weka Segmentation plugin, an ML-based technique within Fiji. The Weka segmentation parameters focused on image texture features, including mean, median, entropy, and variance. Pixel intensity values in each region ranged from 0 to 255. The classifier used in the plugin was trained on OCTA images obtained from one patient included in the study. These images were selected based on clear pattern (vascular/avascular) and consistent signal quality. Three linear regions of interest were drawn in representative areas to train the classifier with the correct boundaries. Once trained, the model was saved and applied uniformly to the entire dataset without further modification. (Figure 2).

To Assess the Agreement Between Algorithms, We Used Bland–Altman Analysis. Pearson’s Correlation Coefficient Was Also Computed to Evaluate Linear Association.

## 3. Results

The study included 59 patients, with a mean axial length of 23.79 ± 1.7 mm and a mean refraction of −0.50 ± 2.64 D. A total of 59 patients who completed the study protocol were included in the final analysis. The mean age was 68.9 ± 7.65 years, with 55.9% female and 44.1% male participants. Eye laterality was balanced, with 29 right eyes and 30 left eyes included. The preoperative spherical equivalent was −0.50 ± 2.64 diopters, and the mean axial length was 23.79 ± 1.7 mm. The best-corrected decimal visual acuity improved from a preoperative mean of 0.69 ± 0.23 to 0.98 ± 0.10 at the final postoperative visit. FAZ measurements across the three vascular plexuses and four visits are summarized in Table 1.

**Figure 3:**
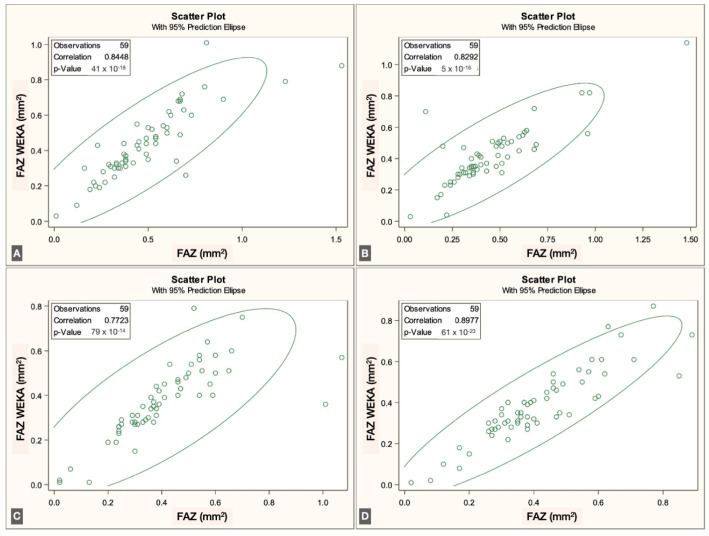
Correlations between ImageJ scripts and ML measurements at the four visits to the SVP (from (**A**–**D**), first to fourth visits).

**Figure 4:**
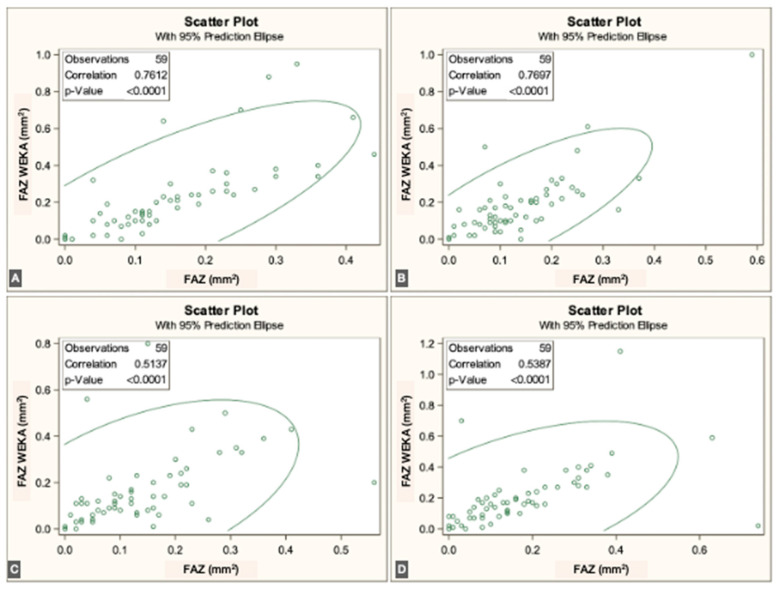
Correlations between ImageJ scripts and ML measurements at the four visits to the IVP (from (**A**–**D**), first to fourth visits).

**Figure 5:**
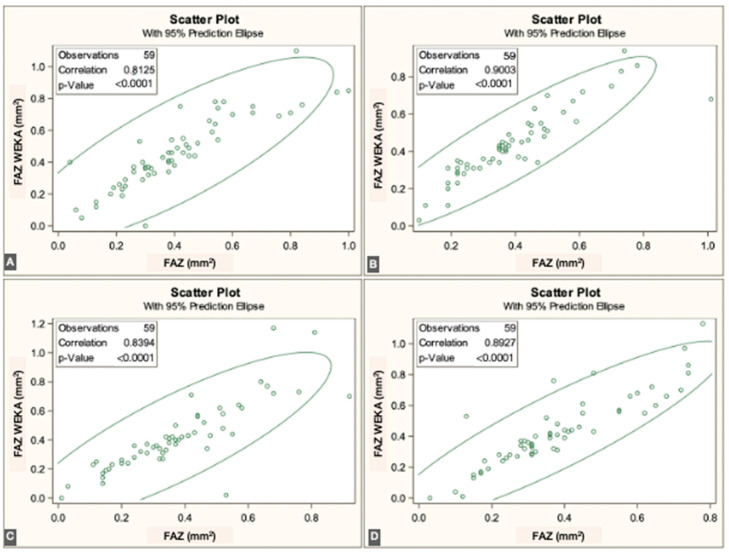
Correlations between ImageJ scripts and ML measurements at the four visits to the DVP (from (**A**–**D**), first to fourth visits).

The FAZ area in the superficial vascular plexus (SVP) was similar between the script-based and ML-based methods [0.44 mm^2^ vs. 0.40 mm^2^; *r* = 0.836 (95% CI: 0.74–0.89), *p* < 0.0001]. Similar agreement was found for the intermediate vascular plexus (IVP) [0.15 mm^2^ vs. 0.19 mm^2^; *r* = 0.646 (95% CI: 0.45–0.78), *p* < 0.0001] and the deep vascular plexus (DVP) [0.39 mm^2^ vs. 0.44 mm^2^; *r* = 0.861 (95% CI: 0.78–0.92), *p* < 0.0001]. Overall, correspondence between methods was strong across all three vascular plexuses. The correlations for each individual visit are illustrated in Figure 3, Figure 4 and Figure 5.

**Figure 6:**
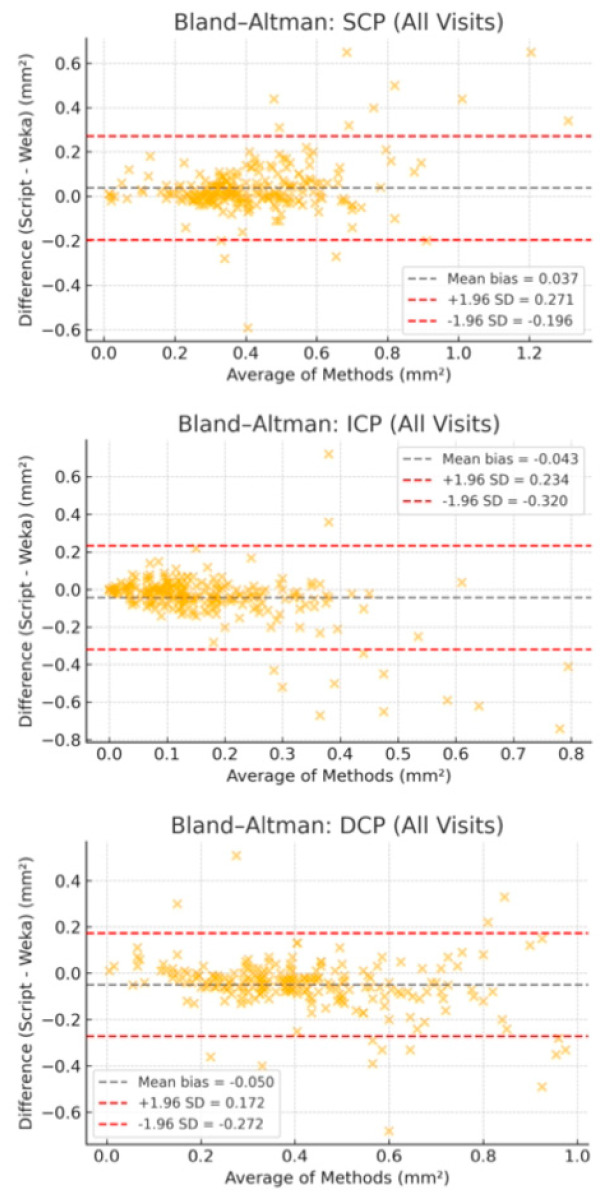
Bland–Altman plots comparing FAZ area measurements obtained using the machine learning−based (Weka) and script-based segmentation methods across three retinal plexuses: SCP (top), ICP (middle), and DCP (bottom), combining all four postoperative time points. The x-axis represents the mean FAZ area between methods, and the y-axis shows the difference (Script–Weka). The solid gray line indicates the mean bias; dashed red lines represent the 95% limits of agreement. All three plots show minimal and systematic bias, confirming overall agreement between the methods despite plexus-specific variability.

In SCP, the Bland–Altman plot revealed a mean difference of 0.010 mm^2^ (95% limits of agreement: [–0.041 to 0.062 mm^2^]). In ICP, the same analysis revealed a mean difference of 0.004 mm^2^ (95% limits of agreement: [–0.036 to 0.045 mm^2^]) and in DCP, 0.007 mm^2^ (95% limits of agreement: [–0.042 to 0.056 mm^2^]), indicating minimal bias between the two segmentation methods. The agreement is illustrated in Figure 6.

**Figure 7:**
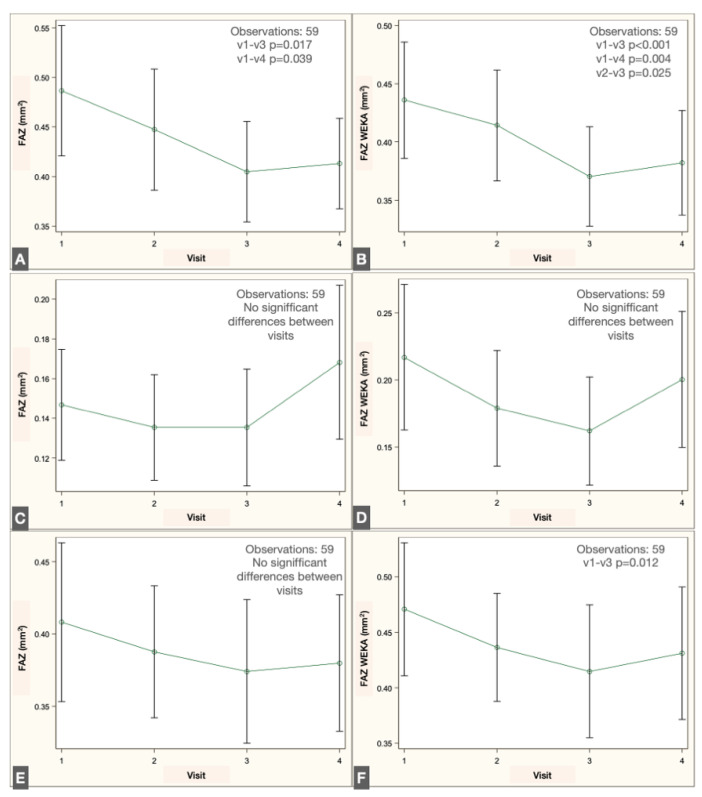
Pre- and postoperative measurements of the FAZ at the four visits in the SVP in ImageJ script (**A**) and ML mode (**B**), in the IVP in ImageJ script (**C**) and ML mode (**D**) and in the DVP in ImageJ script (**E**) and ML mode (**F**) Significative difference between visits are shown in each plot.

Longitudinal analysis revealed a significant reduction in FAZ area within the SVP when comparing the preoperative visit with the 1- and 2-month postoperative visits, both with the script-based method (*p* = 0.017 and *p* = 0.039, repeated-measures linear regression with Tukey adjustment) and the ML-based method (*p* < 0.001 and *p* = 0.004). No significant changes were detected in the IVP using either method. In the DVP, no significant difference was observed across visits with the script-based method; however, the ML-based approach identified a significant decrease in FAZ area between the preoperative and 1-month postoperative visits (*p* = 0.012). The changes in FAZ measurements in the different plexuses are illustrated in Figure 7.

## 4. Discussion

Quantitative analysis of OCTA images is not yet standardized; each manufacturer relies on proprietary software, while non-commercial platforms apply different algorithms to obtain similar outcomes [4]. For example, some OCTA devices incorporate built-in analysis modules, such as the AngioAnalytics™ module in the Optovue RTVue-XR Avanti (Optovue Inc., Fremont, CA, USA), the Angioplex module in the Zeiss Cirrus HD-OCT 5000 (Carl Zeiss Meditec AG, Jena, Germany), and the DRI Triton module from Topcon (Topcon Corporation, Tokyo, Japan) [3]. In contrast, other devices either lack quantitative analysis modules or only offer beta versions that are not widely accessible to the ophthalmic community.

The Spectralis device provides high-resolution images but does not include native tools for quantitative FAZ assessment. In contrast, Fiji software and its available plugins offer versatile solutions for analyzing two-dimensional images. As an open-source image-processing platform specifically designed for scientific applications, Fiji enables reproducible and comparable quantitative analyses through customizable plugins or user-developed scripts. In scientific image analysis, the choice of functions depends on the feature of interest; borders and textures are critical in histologic images and equally relevant in OCTA images.

Both manual and automated approaches to FAZ measurement have been described. Manual delineation requires multiple observers to ensure reproducibility and to account for interobserver variability. However, previous studies have demonstrated substantial inconsistency in manual FAZ measurements, underscoring the need for standardized methods [4].

Several semiautomated scripts have been developed to quantify the FAZ, often implemented in ImageJ macros, Python, or other programming languages [13]. Ishii employed the Li threshold for binarization and applied dilation and erosion masks to refine pixel boundaries [11]. Díaz used the Canny edge detector to establish borders, combined with similar morphological masks to obtain the final segmentation [13]. Gutiérrez-Benítez applied the Ishii script with contrast modifications in a cohort of 29 healthy subjects [14]. In our study, we applied the Otsu threshold for binarization, as it reduces image noise compared with other algorithms [12,15], and used dilation and erosion masks following the approach described by Ishii [11].

Weka, an ML-based plugin developed to differentiate borders and regions by analyzing pixel patterns, has previously been applied to quantify the foveal ellipsoid zone after vascular occlusion [16]. In the present study, we used this Fiji plugin to quantify the FAZ.

The correlation between the two segmentation methods was lower in the intermediate vascular plexus (IVP) compared to the superficial (SVP) and deep (DVP) plexuses. This discrepancy may stem from the anatomical and technical complexity of IVP segmentation. The IVP lies between two densely vascularized layers and often lacks clear boundaries, increasing susceptibility to projection artifacts and segmentation ambiguity. Additionally, image noise and reduced contrast in this layer may challenge the consistency of both algorithmic approaches. These findings suggest that specific segmentation strategies or refined preprocessing may be necessary to achieve accurate and reproducible FAZ measurements in the IVP.

To provide a more accurate evaluation, we performed Bland–Altman analysis pooling all visits for each plexus. The results demonstrated that the IVP exhibited levels of agreement comparable to those of SCP and DCP, with a consistent bias and acceptable limits of agreement. Importantly, no segmentation errors or methodological failures were observed in cases identified as outliers, and discrepancies could be attributed to natural anatomical variability or subtle capillary differences near the FAZ boundary. The analysis revealed a consistent bias, with the script method tending to produce slightly larger FAZ areas than Weka, especially in SCP and DCP. Although some measurements fell outside the 95% limits of agreement, detailed case-by-case inspection showed that most outliers did not reflect segmentation failure but rather natural anatomical variability or known challenges in plexus delineation.

We interpret the divergence as reflecting different algorithmic sensitivities, not artifact detection. The ML-based approach may be better suited to detect subtle vascular remodeling, especially in layers with lower contrast, while the script-based method applies stricter pixel classification thresholds. Rather than undermining the validity of either technique, this difference reinforces the need to interpret FAZ changes in light of the method used, and to apply consistent segmentation strategies in longitudinal analyses.

Therefore, although the methods are not directly interchangeable, they both provide clinically useful and reproducible measurements. Their relative differences are important to acknowledge when interpreting longitudinal FAZ changes or comparing studies using different segmentation strategies.

This study has limitations. First, we did not compare the two automated segmentation methods with manual delineation of the FAZ or with measurements obtained from commercial OCTA analysis software, which limits benchmarking against other references, even though they are not gold standard, since standardization is not available. Second, all data were obtained from a single clinical center using the same OCTA device (Heidelberg Engineering). Third, the patient population was relatively homogeneous in terms of ethnicity and geographic origin. These factors may affect the external validity and generalizability of the results to other populations, devices, or clinical settings. Another limitation of this study is the lack of manual FAZ delineation by expert graders, which could have served as a reference standard to validate the performance of the ML-based segmentation method. Future studies incorporating masked manual grading would be valuable to establish the clinical validity and sensitivity of machine learning segmentation in this context.

FAZ characterization following phacoemulsification has been scarcely reported. Perente investigated changes in macular thickness on OCT after phacoemulsification [17], while Oh described hyperreflective dots in OCT images of patients with macular edema after surgery [18]. Chetrit reported vascular density in OCTA images of eight patients with macular edema post-phacoemulsification [19]. More recently, Liu demonstrated a decrease in FAZ area after phacoemulsification in a cohort of 58 patients, consistent with our findings [20]. Experimental data also support these observations, as increased levels of pro-inflammatory proteins such as chemokine 2 and interleukin-1β have been reported in the neurosensory retina of mice following lens extraction. These molecular changes increase macular capillary permeability and reduce the barrier function of retinal pigment epithelial cells, leading to retinal thickening [21].

Automated FAZ quantification, including the use of artificial intelligence software, is increasingly implemented to improve OCTA analysis. Survey-based studies have attempted to establish normative values for quantitative parameters to facilitate future standardization [4]. In our study, we compared an ImageJ script with the Trainable Weka Segmentation plugin and found that both methods yielded highly correlated results, consistent with prior publications. To our knowledge, this is the first study to apply Trainable Weka Segmentation, an ML-based Fiji plugin, for FAZ quantification in OCTA images.

## 5. Conclusions

The FAZ decreased significantly two months after phacoemulsification in the superficial vascular plexus. The Trainable Weka Segmentation plugin, an ML-based tool, provided reliable and reproducible FAZ measurements, comparable to those obtained with a semiautomated script-based method. These findings support the feasibility of incorporating open-source, AI-based approaches into OCTA analysis to advance the standardization of quantitative retinal imaging.

## Figures and Tables

**Figure 2:**
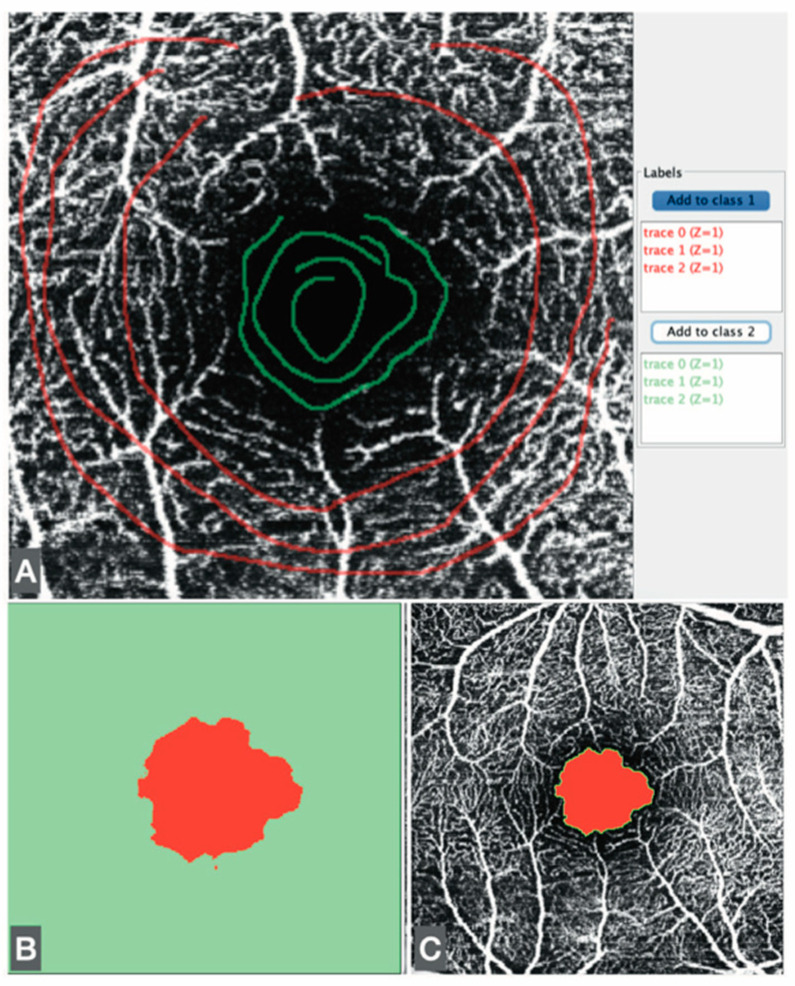
(**A**) Sequences drawn freehand to teach the ML plugin to differentiate zones. (**B**) The results of the ML plugin. (**C**) Fit of the result in the 8-bit former image.

**Table 1:** Area of the FAZ (mm^2^) in the three vascular plexuses, comparing the script-based and ML.

Visit	SVP Script	SVP Weka	IVP Script	IVP Weka	DVP Script	DVP Weka
Visit 1	0.49 ± 0.25	0.44 ± 0.19	0.15 ± 0.11	0.22 ± 0.21	0.41 ± 0.21	0.47 ± 0.23
Visit 2	0.45 ± 0.23	0.41 ± 0.18	0.14 ± 0.10	0.18 ± 0.17	0.39 ± 0.18	0.44 ± 0.18
Visit 3	0.41 ± 0.19	0.37 ± 0.16	0.14 ± 0.11	0.16 ± 0.16	0.37 ± 0.19	0.42 ± 0.23
Visit 4	0.41 ± 0.17	0.38 ± 0.17	0.17 ± 0.15	0.16 ± 0.20	0.38 ± 0.18	0.43 ± 0.23

SVP = superficial vascular plexus, IVP = intermediate vascular plexus, DVP = deep vascular plexus, Script = plexus measured with ImageJ script, Weka = plexus measured with the ML plugin.

## Data Availability

The original contributions presented in this study are included in the article. Further inquiries can be directed to the corresponding authors.

## Abbreviations

The following abbreviations are used in this manuscript:

Abbreviation	Meaning
AI	Artificial Intelligence
DVP	Deep Vascular Plexus
FA	Fluorescein Angiography
FAZ	Foveal Avascular Zone
IVP	Intermediate Vascular Plexus
ML	Machine Learning
OCT	Optical Coherence Tomography
OCTA	Optical Coherence Tomography Angiography
SD	Standard Deviation
SVP	Superficial Vascular Plexus
